# Generation of c-MycER^TAM^-transduced human late-adherent olfactory mucosa cells for potential regenerative applications

**DOI:** 10.1038/s41598-019-49315-6

**Published:** 2019-09-13

**Authors:** Gerardo Santiago-Toledo, Melanie Georgiou, Joana dos Reis, Victoria H. Roberton, Ana Valinhas, Rachael C. Wood, James B. Phillips, Chris Mason, Daqing Li, Ying Li, John D. Sinden, David Choi, Parmjit S. Jat, Ivan B. Wall

**Affiliations:** 1 Department of Biochemical Engineering, University College London, London, WC1H 0AH UK; 2 Aston Medical Research Institute and School of Life & Health Sciences, Aston University, Birmingham, B4 7ET UK; 3 Department of Pharmacology, UCL School of Pharmacy, London, WC1N 1AX UK; 4 UCL Centre for Nerve Engineering, London, WC1E 6BT UK; 5 AVROBIO Inc, Cambridge, MA 02139 USA; 6 Department of Neurosurgery, National Hospital for Neurology & Neurosurgery, London, WC1N 3BG UK; 7 MRC Prion Unit at UCL, Institute of Prion Diseases, London, W1W 7FF UK; 8 ReNeuron Limited, Pencoed, Bridgend CF35 5HY UK; 9 Institute of Tissue Regeneration Engineering (ITREN), Dankook University, Cheonan, 31116 Republic of Korea

**Keywords:** Cell culture, Regenerative medicine, Spinal cord injury

## Abstract

Human olfactory mucosa cells (hOMCs) have been transplanted to the damaged spinal cord both pre-clinically and clinically. To date mainly autologous cells have been tested. However, inter-patient variability in cell recovery and quality, and the fact that the neuroprotective olfactory ensheathing cell (OEC) subset is difficult to isolate, means an allogeneic hOMC therapy would be an attractive “off-the-shelf” alternative. The aim of this study was to generate a candidate cell line from late-adherent hOMCs, thought to contain the OEC subset. Primary late-adherent hOMCs were transduced with a c-MycER^TAM^ gene that enables cell proliferation in the presence of 4-hydroxytamoxifen (4-OHT). Two c-MycER^TAM^-derived polyclonal populations, PA5 and PA7, were generated and expanded. PA5 cells had a normal human karyotype (46, XY) and exhibited faster growth kinetics than PA7, and were therefore selected for further characterisation. PA5 hOMCs express glial markers (p75^NTR^, S100ß, GFAP and oligodendrocyte marker O4), neuronal markers (nestin and ß-III-tubulin) and fibroblast-associated markers (CD90/Thy1 and fibronectin). Co-culture of PA5 cells with a neuronal cell line (NG108-15) and with primary dorsal root ganglion (DRG) neurons resulted in significant neurite outgrowth after 5 days. Therefore, c-MycER^TAM^-derived PA5 hOMCs have potential as a regenerative therapy for neural cells.

## Introduction

Spinal cord injury (SCI) is a devastating condition affecting 250,000 to 500,000 people a year worldwide^1^. The spinal cord is part of the central nervous system (CNS), a tissue with limited regenerative capacity due to the complexity of an unfavourable microenvironment for the re-establishment of neuronal connections^2–5^. In contrast with the peripheral nervous system (PNS), the CNS regenerates slowly due to the formation of “glial scars” that are thought to be biochemical and mechanical barriers for axonal regrowth^6–9^. In the 1980s, studies focused on the ability of olfactory neurons to regenerate and form connections after surgical lesions throughout life^7,10–12^. Such unique regenerative properties have been attributed to the anatomical boundaries of the olfactory system between the PNS and CNS, as well as the presence of cell types such as neural stem cells (NSCs), olfactory ensheathing cells (OECs), and mesenchymal stem cells (MSCs)^13–15^.

The olfactory mucosa is an accessible source of cells for transplantation as biopsies can be safely taken without invasive intracranial surgery unlike the olfactory bulb^16,17^. Moreover, human olfactory mucosa cells (hOMCs) have been successfully transplanted in clinical trials^18–21^. This work has shown few adverse effects from immunological and microbiological perspectives and autologous hOMC transplantation is generally considered safe^22^. However, an autologous process is difficult to standardise due to large variability in cell composition, thus justifying the need to assess an allogeneic or universal “off-the-shelf” approach. Allogeneic cell therapy aligns with the concept of cells as pills^23^, but cells as a product themselves raises challenges for design and standardisation of biomanufacturing processes^24^. According to European Commission guidelines, the sequential generation of master cell banks (MCBs) and working cell banks (WCBs) is suggested for the manufacture of advanced therapy medicinal products (ATMPs)^25^. Nonetheless, this cell-banking expansion model assumes that the cell line is stable and grows indefinitely to achieve a million-fold expansion of the initial stock^26^. Human olfactory mucosa cells have limited lifespans, an issue that compromises the development of a cell-banking model based manufacturing process^27^. Conditional immortalisation technologies aim to overcome this via controllable or inducible genes that, when transduced into cells and under permissive conditions, promote cell proliferation in an immortal state. Under non-permissive conditions, cells lose their immortality and return to a “normal” phenotype^28^. Such an approach has been successfully applied to generate neural stem cell lines from human fetal cortical neuroepithelial cells following retroviral transduction with c-MycER^TAM^ gene^29^. This type of technology consists of a fusion gene encoding a chimeric protein composed of the transcription factor c-Myc and the hormone-binding domain of a mutant murine estrogen receptor (G525R). The gene product of this fusion no longer binds to 17b -estradiol or estrogen-like molecules present in cell culture media, but is responsive to activation by the presence of the synthetic drug 4-hydroxytamoxifen (4-OHT)^30,31^. The expression of the c-MycER^TAM^ protein has been shown to be safe and does not affect the phenotype or the potency of transduced cells^26,32–34^.

In the current work, we present the generation and characterisation of two c-MycER^TAM^-derived hOMCs populations for potential therapeutic application in SCI. First, two independent mucosal biopsies, named as PA5 and PA7, were obtained following informed consent under approval from the ethics committee. To enrich neuroprotective cell types such as OECs, late-adherent primary hOMCs were retrieved by first removing rapidly adherent cells (i.e. fibroblasts) with a 24-hour differential adhesion step. Cells remaining in the supernatant were then re-plated and expanded for 12 days to be transduced by retroviral infection. Stably transduced cells were selected with geneticin (G418), and incorporation of the c-MycER^TAM^ transgene was further validated at DNA and protein level. Karyotype analysis and growth kinetics characterisation was performed for both populations. Due to their karyotypic stability and faster growth kinetics, PA5 hOMCs were chosen for further phenotypic characterisation. Biomarker expression of PA5 hOMCs was assessed by immunocytochemistry to confirm the presence of characteristic glial (p75^NTR^, S100 calcium-binding protein B (S100ß), glial fibrillary acidic protein (GFAP), and oligodendrocyte marker O4), neuronal (ß-III-tubulin and nestin), and fibroblast-associated (Thy1 and fibronectin) markers reported in primary hOMCs^16,35–37^. To mimic a cell contact regeneration process, co-cultures of PA5 hOMC monolayers were performed with the NG108-15 cell line, a hybrid rat glioma mouse neuroblastoma cell line, and with primary dorsal root ganglion (DRG) neurons. In these systems, PA5 hOMCs were able to enhance neurite outgrowth. Clones from polyclonal populations such as PA5 c-MycER^TAM^-derived hOMCs could be further derived, expanded, banked, and screened to generate an allogeneic cell therapy product for the treatment of spinal cord injury.

## Results

### Validation of c-MycER^TAM^ transduction

**Figure 1:**
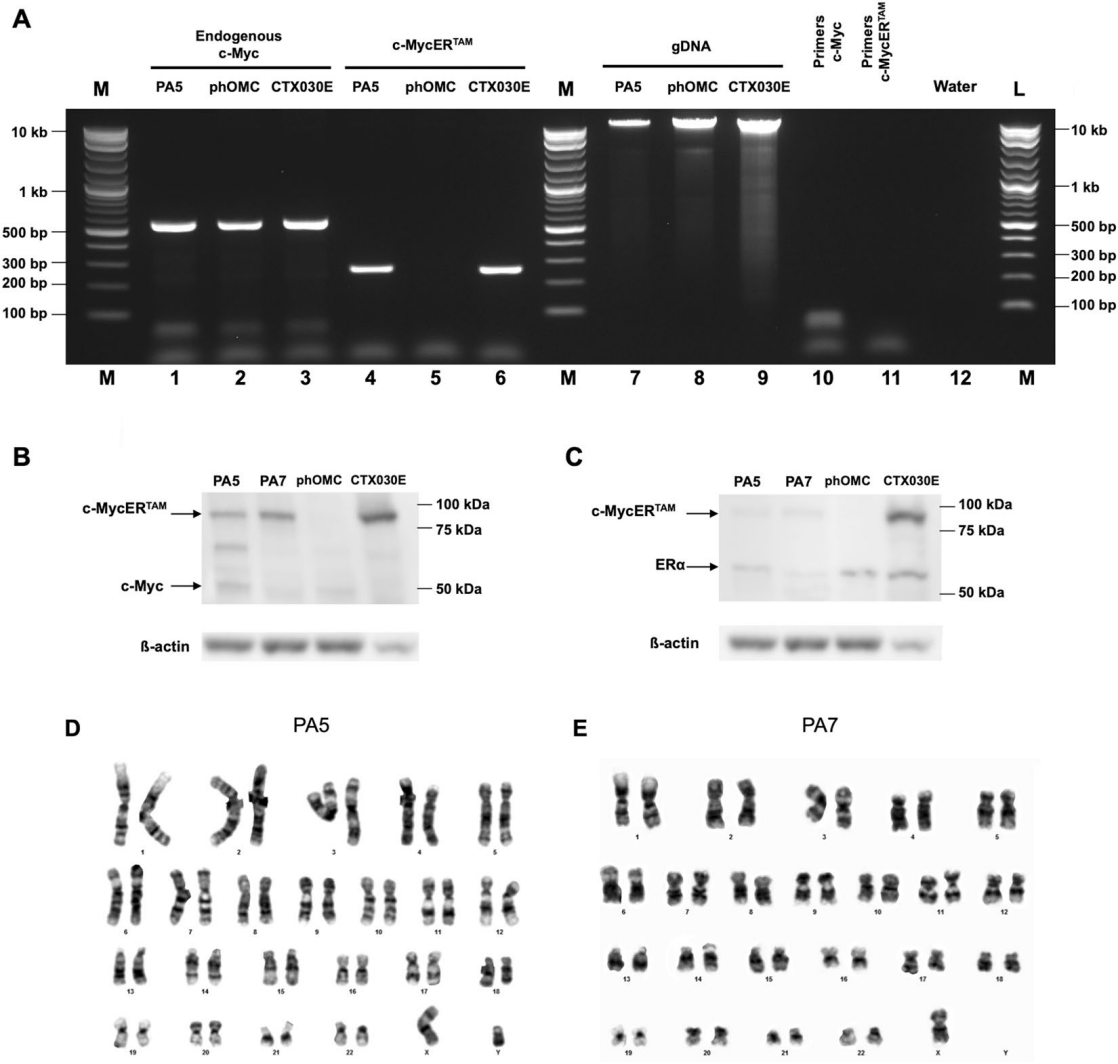
c-MycER^TAM^ transduction characterisation and karyotyping of human olfactory mucosa cells. (**A**) The incorporation of the c-MycER^TAM^ construct in PA5 cells was analyzed by genomic DNA amplification (gDNA) using specific primers (lanes 4–6). CTX0E03 cells were used as a positive control and primary cells from the olfactory mucosa (phOMCs) as a negative control. Amplification of endogenous c-Myc was performed to verify amplification of gDNA samples (lanes 1–3). Finally, gDNA and primers were fractionated to verify integrity (lanes 7–12). Expression of the c-MycER^TAM^ protein is shown by western blotting using (**B**) anti-c-Myc and (**C**) anti-ERα primary antibodies. CTX0E03 cells were used as a positive control and primary cells (phOMCs) as a negative control. (**D**) PA5 cells had a normal 46, XY karyotype, whereas (**E**) PA7 cells had a mosaic karyotype with hypodiploidy (45, X) in 3 of 20 cells.

After selection of stably transduced late-adherent hOMCs with geneticin (G418), successful incorporation of c-MycER^TAM^ to PA5 hOMCs was confirmed at both DNA and protein levels. Genomic insertion of the oncogene was evaluated by amplification of genomic DNA (gDNA) by a standard end-point PCR protocol. Specific primers targeted to the junction of c-Myc and ER^TAM^ were designed to amplify a region of 265 bp. Amplification of endogenous c-Myc was performed to verify amplification of gDNA samples (Fig. 1). Sequences and migration profiles of PCR products of PA5 cells were compared to those of CTX0E03 cells (ReNeuron Ltd.) as a positive control, and primary human olfactory mucosa cells (phOMCs) as a negative control. PCR products were fractionated by agarose electrophoresis, purified and sequenced by Eurofins Genomics. Bands of identical sizes were obtained for both c-Myc and c-MycER^TAM^ targets when amplified using c-Myc specific primers (Fig. 1A lanes 1–3). When amplification was carried out with primers across the junction between c-Myc and ER^TAM^, identical sized bands were obtained for PA5 hOMCs and CTX030E (Fig. 1A lanes 4 and 6), whereas no product was obtained for primary hOMCs (Fig. 1A lane 5). Moreover, sequence alignment yielded 96% to 99% homology between PA5 hOMCs and CTX0E03 c-MycER^TAM^ junction sequences. Western blotting of PA5 and PA7 hOMCs and CTX0E03 lysates revealed that they expressed the c-MycER^TAM^ protein product, in contrast to phOMCs (Fig. 1B,C). The signal from both PA5 and PA7 gene products were of the same size as the CTX0E03 protein with an apparent molecular weight of 90 kDa, distinguishable from endogenous c-Myc (Fig. 1B, band at 55 kDa) and ERα (Fig. 1C, band at 60 kDa). The expression of c-MycER^TAM^ protein for PA5 and PA7 hOMCs was at a lower level relative to CTX0E03 cells. Nonetheless, these results were consistent with gDNA amplification. These analyses confirmed that PA5 cells successfully incorporated the c-MycER^TAM^ fusion gene into their genome, and both PA5 and PA7 hOMCs expressed its respective product at the protein level.

### Karyotype

Incorporation of immortalisation genes has raised concerns about potential karyotypic instability associated with tumori-genesis^38,39^. Moreover, karyotypic abnormalities have been reported in studies of conditional immortalisation of human OB-OECs^40,41^. To determine if chromosomic instability would arise after transduction, standard Giemsa banding (G-banding) karyotypic analysis was carried out for c-MycER^TAM^-derived hOMCs. A representative cell karyotype is shown in Fig. 1D.

PA5 hOMCs at passage 14 (PDL 16.6) displayed a normal diploid male karyotype (46, XY) with no visible chromosomic aberrations, i.e. absence of polyploidy, pseudodiploidy, and hypodiploidy. When the same analysis was performed for PA7 hOMCs at passage 12 (PDL = 16.79); however, these showed a mosaic karyotype as 3 of 20 cells were hypodiploid (45, X) (Fig. 1E).

### Growth kinetics

**Figure 2:**
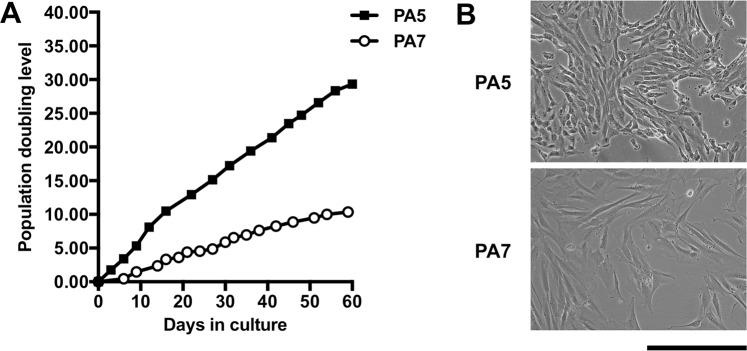
(**A**) Growth of PA5 and PA7 c-MycER^TAM^-derived human olfactory mucosa cells was followed for 60 days. (**B**) Morphology of PA5 and PA7 cells at 100 × total magnification. Scale bar represents 400 µm.

Once the stable incorporation of c-MycER^TAM^ was confirmed, cell growth was monitored manually by performing standard hemocytometer cell counting at every passage for 60 days (Fig. 2A) from passage 4 to 18 for PA5 hOMCs and from passage 9 to 25 for PA7 hOMCs. PA5 hOMCs showed faster growth kinetics than PA7 hOMCs. By day 60, a total of 31.67 cumulative population doublings were observed for PA5 hOMCs, in contrast with 10.34 doublings for PA7 hOMCs. PA5 hOMCs at day 6 were growing at a rate of 1.61 day^−1^ and decreased down to 0.17 day^−1^ at day 60, whereas PA7 hOMCs rates followed a similar pattern from 0.14 day^−1^ at day 6 down to 0.07 day^−1^ at day 60. Thus, doubling times for PA5 hOMCs increased from 0.43 days to 4.05 days, and from 4.88 days to 9.91 days for PA7 hOMCs in the same period. Phase contrast images revealed that PA5 hOMCs at passage 9 had a small, elongated morphology, whereas PA7 hOMCs at passage 10 were larger with higher cytoplasm-to-nucleus ratio (Fig. 2B), a characteristic morphology of senescent cells. After these observations in cell growth, PA5 hOMCs were chosen for further phenotypic characterisation.

### Biomarker expresion of PA5 c-MycER^TAM^-derived hOMCs

**Figure 3:**
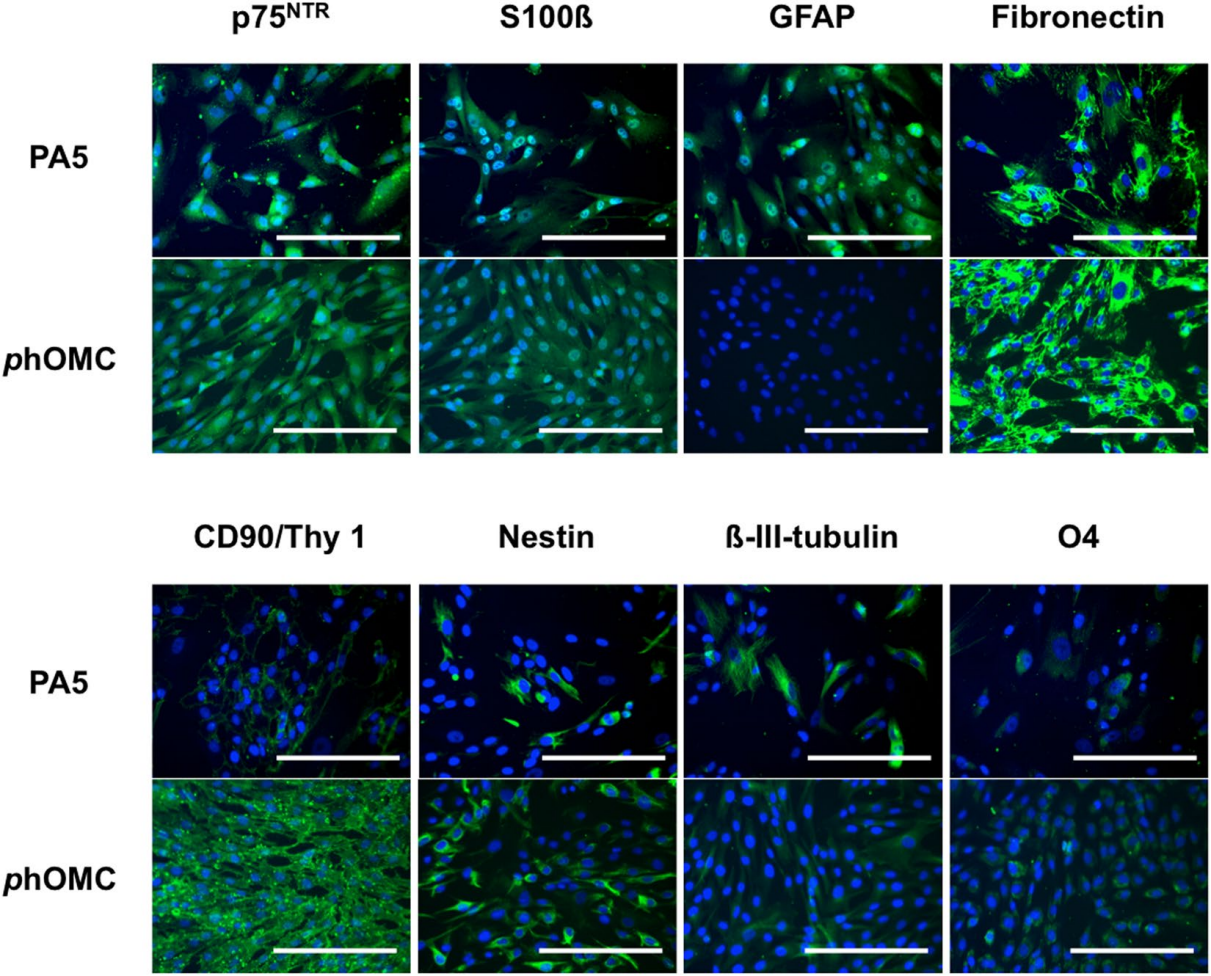
Biomarker expression was assessed by immunocytochemistry for PA5 c-MycER^TAM^-derived hOMCs at passage 8, and primary cells (phOMCs) at passage 3. Both cell populations stained positive for OEC markers (p75^NTR^, S100ß and oligodendrocyte marker O4), neural markers (nestin and ß-III-tubulin), and fibroblast-associated markers (fibronectin, CD90/Thy1) for PA5 and primary hOMCs. Primary hOMCs were negative for GFAP, whereas PA5 hOMCs were positive for this glial marker. Scale bars represent 200 µm.

Initial characterisation of biomarker expression was assessed by immunocytochemistry (ICC) to verify whether PA5 c-MycER^TAM^-derived hOMCs express biomarkers previously reported for cell types present in the olfactory mucosa such as olfactory ensheathing cells (OECs)^16,18,20,42,43^ and mesenchymal stromal cells (MSCs)^15,36,37,44,45^. PA5 hOMCs at passage 8 and phOMCs at passage 3 were stained for OEC markers (p75^NTR^, S100ß, GFAP and oligodendrocyte marker O4), neural markers (nestin and ß-III-tubulin) and fibroblast associated markers (fibronectin, CD90/Thy1) (Fig. 3). Both hOMC populations were positive for tested markers, with the exception of GFAP in phOMCs. Also, a weaker signal for CD90/Thy1 was observed on PA5 hOMCs in comparison with phOMCs. It must be noted that although OM biopsies show intrinsic variability in terms of cell composition^46^, both PA5 hOMCs and phOMCs obtained from different patients, showed similar biomarker profiles. Therefore, this characterisation confirmed that PA5 hOMCs are positive for biomarkers that have been reported in cells isolated from the human olfactory mucosa.

### Co-culture with NG108-15 cells

**Figure 4:**
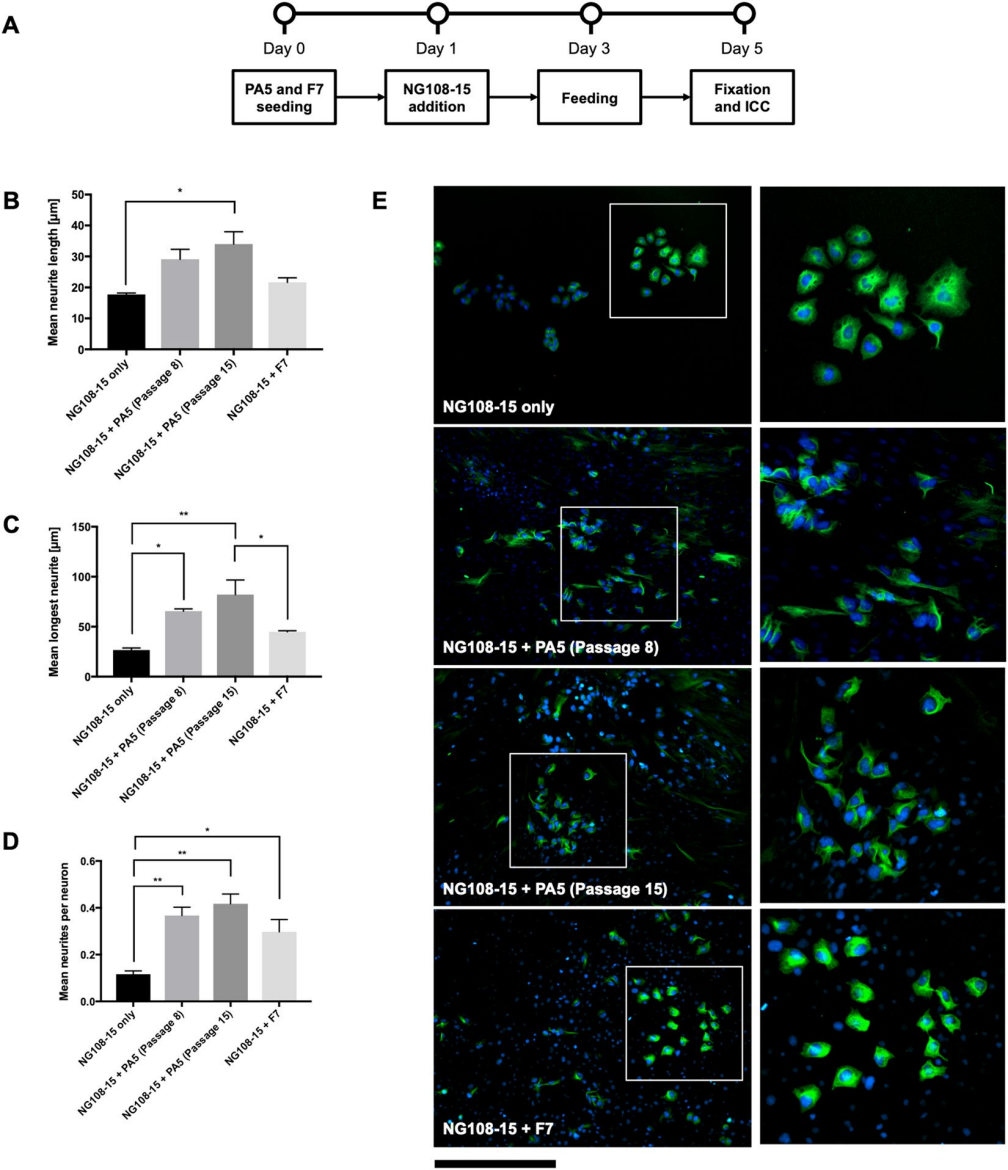
Co-culture of PA5 hOMCs at passage 8 (PDL 10) and passage 15 (PDL 18) with NG108–15 cells. (**A**) Timeline of the co-cultures with NG108-15 cells. (**B**) Mean neurite length, (**C**) mean longest neurite, and (**D**) mean number of neurites per neuron measurements were performed manually. (**E**) Representative images of co-cultures stained with ß-III-tubulin at 100 × total magnification and zoomed regions with NG108-15 cells. Scale bar represents 400 µm at 100 × total magnification, and 200 µm at the zoomed regions. Data are mean SEM, n = 3.

To quantify the potency of PA5 hOMCs, cells were assessed at passage 8 and passage 15 for their ability to support neurite outgrowth. This was assessed using a co-culture assay with hybrid NG108-15 cells. This co-culture assay has been used to investigate the ability of various candidate cell therapies to stimulate NG108-15 cell neurite outgrowth in comparison with a positive control, e.g. Schwann cells^47–50^. Mean neurite length, mean longest neurite, and mean number of neurites per neuron measurements were performed in NeuronJ^51^ by manual tracing of ß-III-tubulin positive neurites in fluorescent images which were clearly distinguishable with immunostaining and morphology from hOMCs (Fig. 4E). Overall, PA5 hOMCs were able to co-exist *in vitro* with NG108-15 cells. The mean neurite length was significantly (H(3, 8) = 9.667, p = 0.0279) higher for NG108-15 cells co-cultured in the presence of PA5 hOMCs in passage 18 (34.01 ± 6.85 µm) when compared to the NG108-15 negative control (17.75 ± 0.75 µm) (Fig. 4B). Among these sprouts, the mean longest neurite was significantly (F(3, 8) = 10.48, p = 0.0038) longer for NG108-15 cells co-cultured with PA5 hOMCs in passage 8 (65.60 ± 3.83 µm), with PA5 hOMCs in passage 18 (82.06 ± 25.24 µm), and with the F7 Schwann cell positive control (44.77 ± 1.99 µm) (Fig. 4C). When calculating the mean ratios of neurites per neuron, they were significantly (F(3, 8) = 11.55, p = 0.0028) higher for NG108-15 cells co-cultured with PA5 hOMCs in passage 8 (0.37 ± 0.06), PA5 hOMCs in passage 18 (0.42 ± 0.07), and the F7 Schwann cell positive control (0.30 ± 0.09) (Fig. 4D). In summary, PA5 hOMCs showed regenerative potential by promoting NG108-15 neurite outgrowth and performed comparably or better than the F7 Schwann cell positive control.

### Co-culture with dorsal root ganglion (DRG) neurons

**Figure 5:**
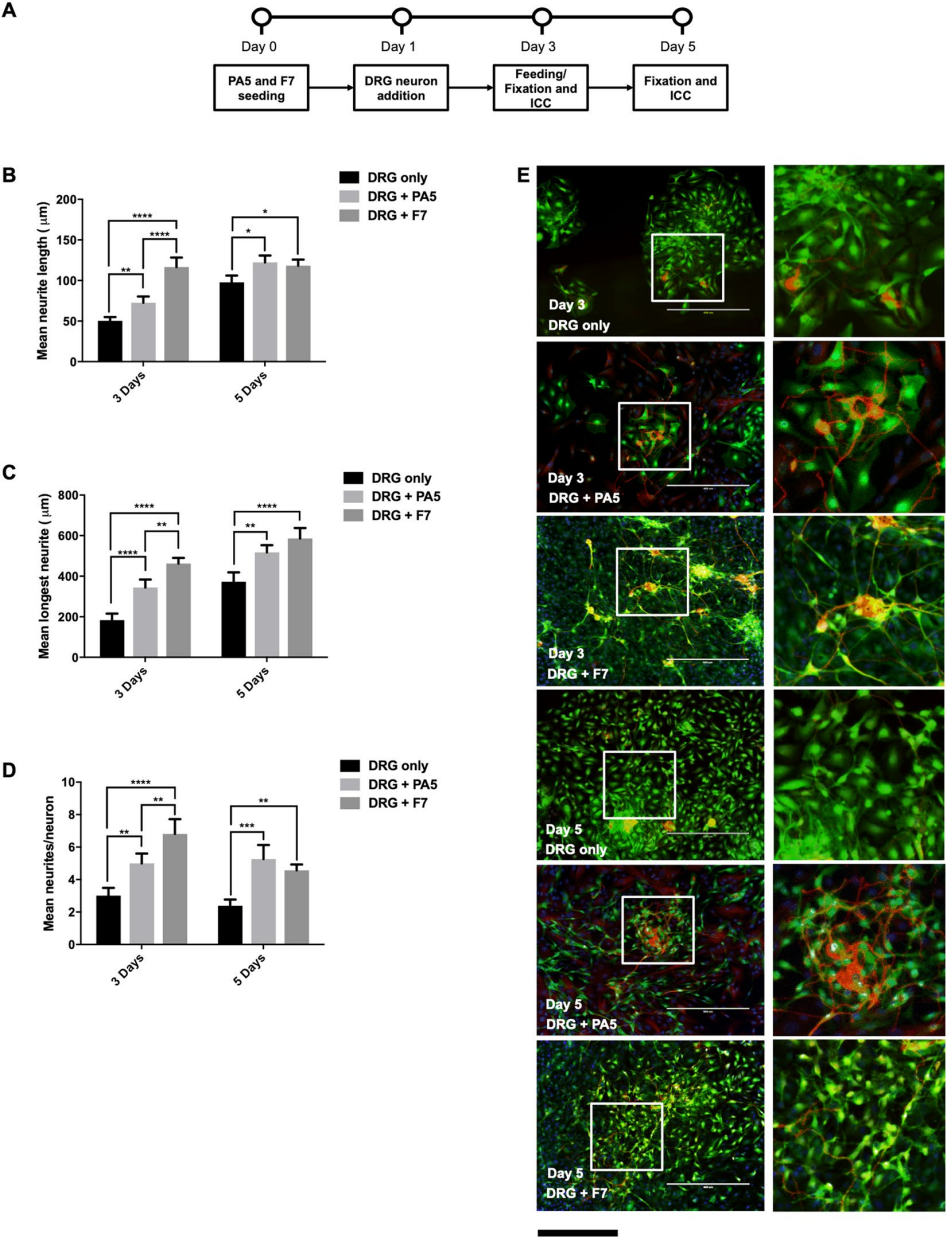
Co-culture of PA5 hOMCs with DRG neurons. **(A**) Timeline of the co-cultures with DRG neurons. (**B**) Mean neurite length, (**C**) mean longest neurite, and (**D**) mean number of neurites per neuron measurements were performed manually. Representative images of co-culture stained with ß-III-tubulin (red) and S100ß (green) at 100 × total magnification and zoomed regions with DRG neurons. Scale bar represents 400 µm at 100 × total magnification, and 200 µm at the zoomed regions. Data are mean SEM, n = 3.

After co-culturing PA5 hOMCs in a cell contact model with NG108-15 cells, PA5 hOMC monolayers were also evaluated using primary rat dorsal root ganglion (DRG) neurons. These were counterstained with specific antibodies targeted to S100ß (green) and ß-III-tubulin after 3 and 5 days of co-culture (Fig. 5). DRG cells co-cultured with F7 Schwann cells had significantly (F(2,12) = 41.06, p < 0.0001) higher mean neurite length (116.60 ± 45.40 µm) than those grown with PA5 hOMCs (72.60 ± 29.7 µm) or with no cells (50.2 ± 18.2 µm) at day 3. However, no differences within PA5 hOMCs and F7 were observed at day 5 (Fig. 5B). Longest neurites were measured for DRG cells grown in the presence of PA5 hOMCs and F7 Schwann cells (Fig. 5C). At both 3 and 5 days of culture, DRG cell co-cultured with F7 Schwann cells and PA5 hOMCs had significantly (F(2,12) = 59.14, p < 0.0001) longer neurites than those cultured alone. At day 3 there was a significant (F(2,12) = 59.14, p = 0.0033) difference of 120 µm between F7 co-culture and PA5 hOMCs. However, the difference of mean longest neurites between F7 and PA5 hOMCs was no longer significant at day 5, indicating that the PA5 hOMCs were equivalent to the positive control.

A significantly (F(2,12) = 36.88, p < 0.0001) higher number of sprouts per cell were quantified on DRG neurons grown with F7 Schwann cells at day 3 (6.81 ± 3.53) and day 5 (4.57 ± 1.37) (Fig. 5D). Interestingly at day 5, DRG cells co-cultured with PA5 hOMCs had the highest number of neurites per cell (5.26 ± 3.38), slightly higher than those grown with F7 Schwann cells (4.57 ± 1.37). These co-culture trends show that PA5 hOMCs also promote neurite outgrowth from rat DRGs. This process seems to be slower for DRG neurons co-cultured with PA5 hOMCs than those grown with F7 cells; however, it provides additional evidence to support the suitability of PA5 hOMCs for promoting neurite outgrowth in the damaged spinal cord.

## Discussion

Although autologous human olfactory mucosa cells (hOMCs) have been successfully transplanted into the damaged spinal cord, as yet no study has achieved successful expansion of this cell population to provide an allogeneic source of cells for transplantation. The pressure to generate cell lines as biomedical research tools has triggered the development of approaches for the production of stable and well characterised cells. Initially cell lines were obtained from tumours or spontaneous variants that grew readily in culture^28,52,53^. It was not until the development of vectors for insertion of desired genes into the genome that the generation of immortalised cells that were not derived from tumours became possible. Expression of immortalisation genes raises safety concerns due to them conferring an unlimited proliferative potential, which may lead to tumorigenesis^54^. There are consequently a number of challenges to be addressed for conditional immortalisation technologies, most of them safety related (e.g. transgene stability, controlability, silencing prior to transplantation). Nonetheless, the success of cases such as CTX0E03 by ReNeuron Ltd. has opened R&D opportunities across the cell therapy industry to generate commercially sustainable manufacturing processes^28^. Conditional immortalisation technologies have the potential to produce enough cells for the generation of MCBs and WCBs.

In this study, we successfully incorporated a c-MycER^TAM^ conditional immortalisation gene into the genome of two polyclonal populations of hOMCs via retroviral transduction. Late-adherent hOMCs were chosen based on the fact that fibroblasts attach to uncoated polystyrene surfaces rapidly^55^ (<one hour), whereas other cell types linked to regeneration (such as olfactory ensheathing cells) do not attach within the first 24–48 hours^35,56^. It is therefore possible to enrich these candidate regenerative cells by selectively removing mucosal fibroblasts based on adhesion properties. After selection of stably transduced cells, the incorporation of the transgene was verified at DNA and protein levels. Both c-MycER^TAM^-derived populations incorporated the transgene into their genome and expressed the c-MycER^TAM^ gene product. In the first thirty days of culture, a 40-fold expansion in PA5 hOMC numbers was achieved, with progressive decrease in growth kinetics. Transduction of genes under the cytomegalovirus (CMV) immediate early promoter has been performed to successfully generate CTX0E03 neural stem cells^29^. Interestingly, it has been shown that CMV promoters can undergo epigenetic silencing^32^, and can be either strong or weak to express proteins in particular cell types^57,58^. As the signal of c-MycER^TAM^ protein for PA5 hOMCs was weaker when compared to the CTX0E03 control, it might be possible that silencing events had been occurring in long-term culture, which remain to be further characterised. Nonetheless, PA5 hOMCs were further investigated for phenotypic characterisation due to their faster growth kinetics and karyotype stability when compared to PA7 hOMCs or to other published reports of conditionally immortalised glia from the olfactory bulb^40,59,60^. PA5 hOMCs expressed glial markers (p75^NTR^, S100ß, oligodendrocyte marker O4), neuronal markers (nestin, ß-III-tubulin), and fibroblast-associated markers (fibronectin, CD90/Thy1) reported in cell types isolated from olfactory mucosa such as olfactory ensheathing cells^16,18,20,42,43^, and more recently, mesenchymal stem cells^15,36,37,44,45^ from the lamina propria. Moreover, a potency assay using a co-culture model based on stimulation of axonal growth in NG108-15 hybrid cells revealed that PA5 cells had promising functional activity. Previous PNS repair studies using this co-culture model have shown that neurite outgrowth *in vitro* can be consistently associated with Schwann cell potency to enhance neurite length *in vivo*^47,48^. The effect of PA5 cells on neurite outgrowth was comparable to F7 Schwann cells, assessed using several neurite outgrowth parameters. Measurements of mean number of neurites per neuron, mean length and mean longest length were higher overall for PA5 hOMCs than for the F7 Schwann cell positive control, demonstrating that PA5 hOMC monolayers promoted neurite outgrowth of NG108-15 cells at two separate passages. A similar effect on neurite growth was observed when primary rat DRG neurons were used in the co-culture model instead of the NG108-15 cell line. This confirmed the ability of PA5 hOMCs to support neuronal cell activity *in vitro*. These preliminary co-culture data are promising, however future studies using *in vivo* models will be necessary to determine whether cell transplantation into damaged spinal cord can lead to functional recovery. They will also be important to ensure that the gene-modified cells have an acceptable safety profile, similar to that seen in earlier c-MycER^TAM^-transduced cells^28,29^.

In summary, we demonstrate proof-of-principle for increased expansion of hOMCs using c-MycER^TAM^ technology to produce a population of cells that could be used to expand cells for transplantation, and further separated to select clones with desirable regenerative properties. This study provides early evidence that late-adherent, c-MycER^TAM^-derived hOMCs are a promising candidate for supporting neural regeneration. This approach may underpin the development of an off-the-shelf advanced therapy medicinal product for the treatment of CNS injuries such as those of the spinal cord.

## Methods

### Primary culture

**Figure 6:**
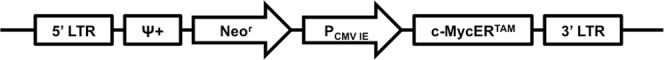
Structure of the c-MycER^TAM^ provirus.

Adult olfactory mucosa biopsies were taken in accordance to regulatory requirements following informed consent under approval from the NHS Research Ethics Service (REC reference 18/LO/0108, IRAS project ID 229297) at the National Hospital for Neurology and Neurosurgery. All methods were performed in accordance with the relevant guidelines. Biopsies were taken and primary culture was performed^46^. After surgery, tissue was transported in DMEM/F12 (Gibco, Life Technologies) + GlutaMAX^TM^-I (Gibco, Life Technologies) + 10% FBS (Sigma) + 1% penicillin/streptomycin (P/S) medium to the laboratory, and allowed to rest for 2 hours. The olfactory mucosa (OM) was dissected and chopped using a scalpel (TAAB). Sliced OM was rinsed in Hank’s Balanced Salt Solution (HBSS, Gibco, Life Technologies) to remove mucus and conditioned with fresh FBS expansion medium. Rinsed OM was then cut into small pieces of approximately 0.5 mm^2^, transferred to 15-mL centrifuge tubes and incubated in 2 mL of dispase II (Boehringer, 2.4 U/mL in FBS expansion medium) at 37 °C for 45 minutes. Dissociated cells were centrifuged (400 × g for 5 min, RT), and transferred to a new tube with collagenase Type H from Clostridium histolyticum (Sigma, 0.0025 g/mL in FBS expansion medium). Primary human olfactory mucosa cells (phOMCs) were incubated at 37 °C for 15 minutes, and mechanically triturated every 5 minutes. After enzymatic dissociation, polyclonal populations of phOMCs were plated onto Petri dishes, and maintained in standard culture conditions at 37 °C, 5% CO_2_. A differential adhesion step was carried out 24 hours later to remove the rapidly adhering fibroblasts, allowing cell types with low adhesion rates (>24 hr) such as OECs to be enriched in the subsequent culture^35^. Late-adherent hOMCs in the supernatant were re-plated onto a new Petri dish pre-coated with poly-L-lysine (PLL, Sigma, 100 µg/mL). Cells were maintained in standard culture conditions at 37 °C, 5% CO_2_, and fed every 48 hours for 12 days prior to transduction with the c-MycER^TAM^ retrovirus (Fig. 6).

### c-MycER^TAM^ transduction

**Figure 7:**
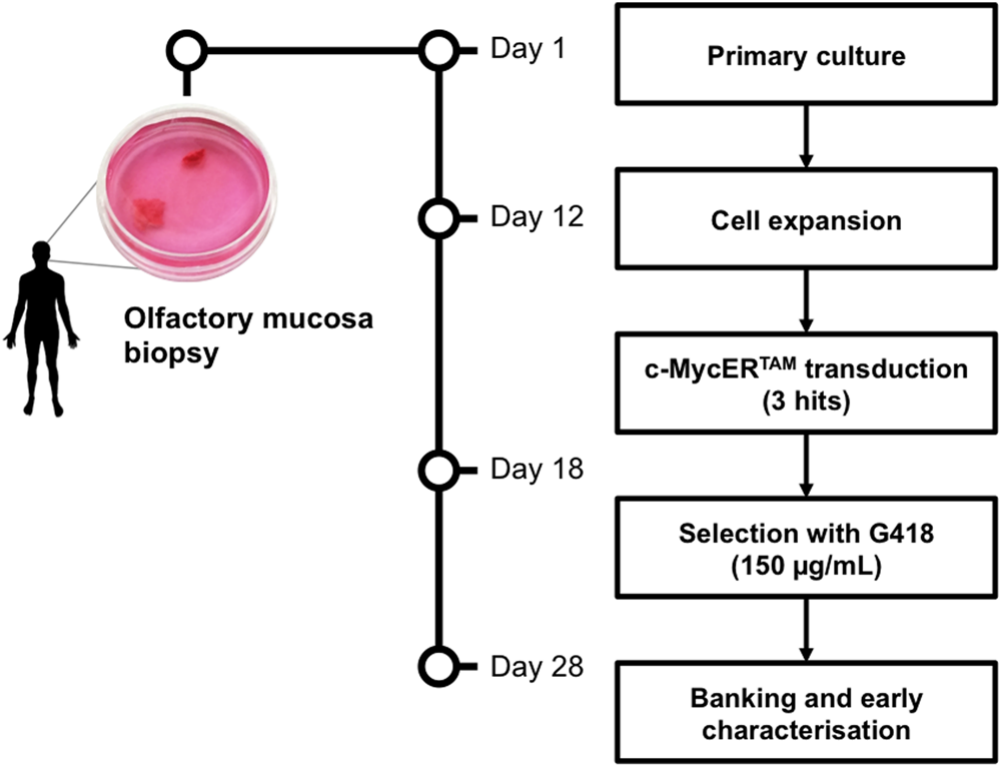
Generation of c-MycER^TAM^ - derived populations of human olfactory mucosa cells (hOMCs).

Cell populations were generated by viral transduction of primary human olfactory mucosa cells with the c-MycER^TAM^ fusion gene (Fig. 7). Briefly, c-MycER^TAM^ construct cloned into pLNCX-2 was packaged as an amphotrophic virus using the TEFLY-A packaging cell line^29^. Virus was harvested in DMEM/F12 (Gibco, Life Technologies) + GlutaMAX^TM^-I (Gibco, Life Technologies) + 10% FBS (Sigma) + 1% penicillin/streptomycin (P/S, Sigma) medium over an 8 hour period and filtered through a 0.45 mm filter (Millipore) to remove cell debris. Primary human olfactory mucosa cells (hOMCs) were expanded for 12 days after surgery, and exposed to viral supernatants in the presence of polybrene (4 mg/mL) for 8 hours. Subsequently, cells were fed with fresh expansion medium, and viral infections were carried out sequentially three times at intervals of 16 hours to maximise the efficiency of stable viral transduction. After the infections, cells were exposed to geneticin (G418, 150 µg/mL) to select stably transduced cells. Non-infected primary hOMCs were also exposed to G418, and when no cells remained in the untreated dish by day 10, G418-resistant hOMCs were banked and further characterised. Two c-MycER^TAM^-derived polyclonal populations, named PA5 and PA7, were used in this study.

### Cell culture

PA5 and PA7 hOMCs were grown in complete expansion medium (DMEM/F12 (Gibco, Life Technologies) + GlutaMAX^TM^-I (Gibco, Life Technologies) + 10% FBS (Sigma) + 100 nM 4-OHT (Sigma)). When cells reached 60–80% confluency, they were washed with HBSS, and passaged using TryPLE^TM^ (Gibco, Life Technologies) for 6 min at 37 °C. After incubation, dissociation reagent was stopped by dilution in expansion media. The cell suspension was centrifuged (400 × g for 5 min, RT) and cells resuspended in a known volume of media. Cells were counted by hemocytometer, plated at a seeding density of 6,000 cells/cm^2^, and maintained in standard culture conditions at 37 °C, 5% CO_2_, with feeding every 48 hours.

### Manual cell counting

Cells were counted using a Bright-Line^™^ (Reichert, NY, USA) hemocytometer under a Life Technologies EVOS^®^ inverted light microscope. 10 mL of cell suspension were applied to each chamber. Twelve squares were manually counted and averaged (Eq. 1).

1 $${n}_{cells/mL}=\frac{1}{{n}_{sq}}\mathop{\sum }\limits_{i=1}^{{n}_{sq}}{n}_{i}\times {10}^{4}$$

where

n_sq_: Number of squares; and n_i_: Number of cells on each square.

Population doubling level (PDL) was calculated (Eq. 2) to estimate the age of the culture based on the total number of times cells that have divided, and also taking into account cell yield and seeding number^60^.

2 $$PD{L}_{i}=\frac{1}{\mathrm{ln}(2)}\,\mathrm{ln}(\frac{{N}_{fi}}{{N}_{0i}})+PD{L}_{i-1}$$

where

PDL_i_: Final population doubling level; PDL_i−1_: Initial population doubling level; x_fi_: Final cell number; and x_0i_: Initial cell number.

Data were recorded in Microsoft Excel files while passaging and analysed by GraphPad Prism^®^ 7.

### Karyotyping

Cells were expanded until 60–80% confluence was reached. Live cells were shipped to Cell Guidance Systems Ltd. (Cambridge, UK) for karyotype analysis. Briefly, samples of 20 random cells were picked, fixed and stained. Additionally all cells used in this work were routinely tested for mycoplasma every 6 months (Surrey Diagnostics, Cranleigh, UK).

### PCR

Genomic DNA (gDNA) was extracted using a Blood and Cell Culture mini-kit (QIAGEN). Adherent cells were harvested by centrifugation and excess medium was removed prior to storage at −80 °C. gDNA samples were eluted in 70% (% v/v) ethanol, centrifuged and finally diluted in 100 µL of TE buffer (Sigma). Samples were quantified in ng/mL by measuring 260/280 nm absorbance ratios on a NanoDrop ND-1000 spectrophotometer (Thermo Scientific). 100 ng of gDNA template was mixed with a Q5^®^ High-Fidelity (New England Biolabs) Master Mix, DNAse-free water and specific PCR primers (Table 1) for a 25 mL reaction in a T100^TM^ Thermal Cycler (Bio-Rad). PCR products were run on 2% agarose gels in TBE buffer (Gibco, Life Technologies) stained with 0.5 µg/mL ethidium bromide (Sigma). Bands were imaged in a GelDoc 2000 device (Bio-Rad).

**Table 1:** Primers for gDNA amplification.

Target	Primer	Sequence (5′-3′)	Amplicon size	T_m_ (°C)
c-MycER^TAM^	Forward	GAAAAGGCCCCCAAGGTAGT	265 bp	65.1
c-MycER^TAM^	Reverse	TCTGGTCAGCTGTCAAGGAC	265 bp	63.2
Endogenous c-Myc	Forward	GGACTTGTTGCGGAAACGAC	581 bp	59.4
Endogenous c-Myc	Reverse	GGGAGGGGGAAGAAACGAAA	581 bp	59.4

### Immunocytochemistry (ICC)

PA5 hOMCs were grown in T-flasks and re-plated onto PLL-coated well plates at 6,000 cells/cm^2^. Cells were washed with PBS and fixed overnight with 4% paraformaldehyde (PFA, Sigma) at 4 °C. Subsequently they were permeabilised with 0.1% Triton X-100 (Sigma) for 20 min at RT, and blocked with 5% goat serum (Dako) for 30 min at RT. Samples were incubated with primary antibody (1:200, Table 2) for 90 minutes at RT, followed by incubation with secondary antibody (1:200, Table 2) and Hoechst 33342 (1:3000, Invitrogen) for 45 minutes at RT. PBS washes were performed three times for 5 minutes between each step. Five pictures per well were taken under an Eclipse TE2000-U microscope (Nikon) at 100 and 200 total magnification.

**Table 2:** Primary and secondary antibodies for immunocytochemistry (ICC).

Host	Antibody	Reactivity	Company	Code
Rabbit	IgG polyclonal	p75^NTR^	Millipore	ab105389
Rabbit	IgG polyclonal	S100ß	Dako	Z0311
Rabbit	IgG polyclonal	GFAP	Dako	Z0334
Rabbit	IgG monoclonal	Nestin	Millipore	MAB5326
Mouse	IgG monoclonal	CD90/Thy1	Millipore	MAB1406
Mouse	IgG monoclonal	ß-III-tubulin	Sigma	T8660
Mouse	IgG monoclonal	Fibronectin	Sigma	F6140
Mouse	IgG monoclonal	O4	Millipore	MAB345
Goat	IgG DyLight 488	Rabbit IgG (H + L)	Thermo Scientific	35552
Goat	IgG DyLight 488	Mouse IgG (H + L)	Thermo Scientific	35503

### SDS-PAGE/Western blot

Reducing buffer (26% glycerol, 10% ß-mercaptoethanol (BME), 0.34 M Tris (pH = 7.3), and 0.1 g/mL sodium dodecyl sulfate) was added to the samples. Reduction was performed at 95 °C for 10 minutes, and equal amounts of protein were fractionated by SDS-polyacrylamide gel electrophoresis (PAGE) with 4–20% gels (Bio-Rad). The proteins were transferred to PVDF membranes, blocked for one hour with 5% milk in 0.5 TBS + 0.05% Tween 20 (TBST) buffer, and incubated for 16 hours at 4 °C with primary antibody (1:200 in 5% milk in 0.5 TBST, Table 3). After rinsing, the blots were incubated for 2 hours at 4 °C with secondary antibody (1:2000 in 5% milk in 0.5 TBST, Table 3) conjugated with HRP. Membranes were rinsed 5 times with 0.5 TBST between each step. Finally, blots were developed by rinsing for 2 minutes with fresh SuperSignal^™^ West Femto Maximum Sensitivity ECL Substrate (Thermo Scientific) in an Amersham Imager 600 (GE Healthcare).

**Table 3:** Primary and secondary antibodies for western blotting (WB).

Host	Antibody	Reactivity	Company	Code
Mouse	IgG monoclonal	c-Myc	Santa Cruz	sc-42
Rabbit	IgG polyclonal	ER	Santa Cruz	sc-542
Mouse	IgG monoclonal	ß-actin	Abcam	ab8226
Goat	IgG-HRP	Rabbit IgG (H + L)	Abcam	ab6721
Goat	IgG-HRP	Mouse IgG (H + L)	Abcam	ab6789

### Co-culture with NG108-15 cells

Co-cultures in the first stage of this study used established cell lines rather than primary cells to avoid variability in cell populations. PA5 hOMCs were co-cultured with NG108-15 cells (ATCC^®^ HB-12317^®TM^), a hybrid rodent glioma-neuroblastoma cell line^61^ (Fig. 4A). NG108-15 cells were expanded on T-75 flasks (Thermo-Scientific) freshly coated with poly-L-lysine (PLL, Sigma, 100 µg/mL) for two passages according to supplier protocols in DMEM/F12 (Gibco, Life Technologies) + GlutaMAX^TM^-I (Gibco, Life Technologies) + 10% FBS (Sigma) medium. The rat SCL 4.1/F7 (ECACC 93031204) Schwann cell line was used as a positive control^62^. Similarly to NG108-15 cells, F7 cells were expanded on T-75 flasks with no coating for two passages according to supplier protocols in DMEM/F12 (Gibco, Life Technologies) + GlutaMAX^TM^-I (Gibco, Life Technologies) + 10% FBS (Sigma) medium. To start the co-cultures, PA5 and F7 cells were cultured, and re-plated at 6,000 cells/cm^2^ onto 24-well plates (Thermo-Scientific) freshly coated with PLL (Sigma, 100 µg/mL). 4-OHT, which activates the c-MycER^TAM^ conditional immortalisation system, was removed from PA5 hOMC media during cell seeding. After 24 hours, NG108-15 cells were detached and plated at a seeding density of 500 cells/well onto 24-well plates with monolayers of PA5 or F7 cells, and onto negative control wells with NG108-15 cells only. Cells were fed with DMEM/F12 (Gibco, Life Technologies) + GlutaMAX^TM^-I (Gibco, Life Technologies) + 10% FBS (Sigma) every 48 hours, and fixed after 5 days of co-culture. Immunocytochemistry was carried out as described previously. A total of 15 frames were taken per condition (5 per technical triplicate) at 100 × total magnification in the EVOS^®^ FL Imaging system (Thermo-Scientific). Neurite quantification was performed manually in ImageJ using the NeuronJ plugin^51^. Data were recorded in Microsoft Excel files and analysed by GraphPad Prism^®^ 7.

### Isolation of dorsal root ganglion (DRG) neurons

All animal tissue was obtained according to the UK Home Office regulations following approval by the UCL Research Ethics Committee. To obtain the dorsal root ganglia (DRGs), first the spinal column was excised from adult Sprague-Dawley rats (250–350 g) that were culled using carbon dioxide (CO_2_) asphyxiation. The column was divided in half in the sagittal plane to expose the spinal cord, and the cord tissue was removed to expose the DRGs in the intervertebral foramen. Under a dissecting microscope, the DRGs were removed and placed in a petri dish containing growth medium. Twenty DRGs were collected from the thoracic and lumbar regions, and cleaned by removal of the roots, capsule and capillaries. The cleaned DRGs were dissociated after incubation in 2 mL 0.125% collagenase type IV (prepared in basal medium supplemented with 100 mg/mL penicillin/streptomycin solution) at 37 °C, 5% CO_2_ in air for 90 minutes. The collagenase-treated explants were mechanically dissociated (triturated) with a 1 mL pipette. Collagenase was removed by two 20 mL spin washes in growth medium at 400 × g for 5 minutes. The pellet was resuspended in growth medium supplemented with 0.01 mM cytosine arabinoside (to deplete the dividing satellite glial cells and any fibroblasts, leaving an enriched primary culture of adult rat neurons), and plated in a poly-L-Iysine (PLL)-coated (50 µg/mL, RT, 30 minutes) T-75 flask (1–2 rats per flask) and incubated at 37 °C, 5% CO_2_ for 24 hours before use. They were removed by trypsinisation with 0.25% trypsin/EDTA solution for 10 minutes at 37 °C, 5% CO_2_. Cells were recovered by centrifugation at 400 × g for 5 minutes and then resuspended in growth medium for co-cultures.

### Co-culture with dorsal root ganglion (DRG) neurons

In the next stage, PA5 hOMCs were co-cultured with primary human DRG neurons (Fig. 5A). DRG neurons were expanded on T-75 flasks (Thermo-Scientific) freshly coated with poly-L-lysine (PLL, Sigma, 100 µg/mL) for two passages in DMEM/F12 (Gibco, Life Technologies) + GlutaMAX^TM^-I (Gibco, Life Technologies) + 10% FBS (Sigma) medium. To start the co-cultures, PA5 and F7 cells were cultured, and re-plated at 6,000 cells/cm^2^ onto 24-well plates (Thermo-Scientific) freshly coated with PLL (Sigma, 100 µg/mL). 4-OHT, which activates the c-MycER^TAM^ conditional immortalisation system, was removed from PA5 hOMC media during cell seeding. After 24 hours, DRG neurons were detached and plated at a seeding density of 500 cells/well onto 24-well plates with monolayers of PA5 or F7 cells, and onto negative control wells with DRG neurons only. Cells were fed with DMEM/F12 (Gibco, Life Technologies) + GlutaMAX^TM^-I (Gibco, Life Technologies) + 10% FBS (Sigma) every 48 hours, and fixed after 3 and 5 days of co-culture. Immunocytochemistry was carried out as described previously. A total of 15 frames were taken per condition (5 per technical triplicate) at 100 total magnification in the EVOS^®^ FL Imaging system (Thermo-Scientific). Neurite quantification was performed manually in ImageJ using the NeuronJ plugin^51^. Data were recorded in Microsoft Excel files and analysed by GraphPad Prism^®^ 7.

### Statistical analysis

Statistical analyses were conducted with one-way ANOVA followed by Holm-S´ıdak´ post hoc test when data were normally distributed, or non-parametric Kruskal-Wallis H Test by ranks followed by Dunn’s post hoc test when data did not pass an integrated D’Agostino’s omnibus K^2^-Pearson normality test. Data were analysed using GraphPad Prism^®^ 7. For all tests, *p 0.05, **p < 0.01, ***p < 0.001 and ****p < 0.001 were considered to be statistically significant.
